# A case of pericarditis caused by *Mycoplasma hyorhinis* in a weaned piglet

**DOI:** 10.1186/s40813-021-00211-4

**Published:** 2021-04-12

**Authors:** Martina Ustulin, Erica Rossi, Denis Vio

**Affiliations:** 1 Peripheral Diagnostic Laboratory of Pordenone, Istituto Zooprofilattico Sperimentale delle Venezie, Cordenons, PN Italy; 2 Veterinary Practitioner, Montebelluna, TV Italy

**Keywords:** *Mycoplasma hyorhinis*, Pericarditis, Antibiotic resistance

## Abstract

**Background:**

*Mycoplasma hyorhinis* (*M. hyorhinis*) is a bacterium commonly found in the upper respiratory tract of healthy pigs and an agent of polyserositis and polyarthritis. Moreover, it can carry antibiotic resistance genes (Wu et al, Vet. Microbiol. 76: 25–30, 2000). Economic losses caused by *M. hyorhinis* can be reduced by antibiotic therapy, however, isolation and antimicrobic susceptibility profile are rarely performed.

**Case presentation:**

The present report describes a case of pericarditis caused by *M. hyorhinis* in a weaned piglet with respiratory symptoms and reduced growth performance.

At post mortem examination, the main macroscopic finding was a severe fibrinous pericarditis and *M. hyorhins* was the only agent isolated from the pericardial fluid.

In this strain, Minimum Inhibitory Concentration (MIC) determination revealed resistance to various antimicrobial molecules such as erythromycin, tylosin and tilmicosin.

**Conclusion:**

This paper highlights the importance of including *M. hyorhins* in the differential diagnosis of polyserositis in swine. Moreover, due the possible presence of multidrug resistance, the determination of antimicrobial susceptibility pattern should be performed on a regular basis.

## Background

*Mycoplasma hyorhinis (M. hyorhinis)* is frequently found in the upper respiratory tract of swine [3] however, under certain conditions, it can cause systemic infection characterized by arthritis, polyserositis, conjunctivitis and otitis [10]. *M. hyorhinis* can also act as a secondary pathogen in cases of pneumonia [9] caused by Porcine Reproductive and Respiratory Syndrome Virus (PRRSV) and Porcine Circovirus type 2 (PCV2) [4].

The isolation of *M. hyorhinis* requires specific technical expertise and prolonged culture in a specific medium. The final identification is therefore time consuming and the antibiotic susceptibility evaluation is not routinely performed [7].

In this respect, since in Europe an effective vaccine against *M. hyorhinis* is not commercially available yet, the antibiotic treatment is usually performed to reduce the economic impact of this infection. However, recent data on the antimicrobial susceptibility of *M. hyorhinis* European field strains are scarce in the literature [1]. Therefore, the determination of the antibiotic susceptibility profile of *M. hyorhinis* strains associated with clinical disease is essential for the choice of the appropriate antimicrobial therapy.

## Case presentation

During July 2019 a 40-day-old weaned piglet was found dead and sent to the Diagnostic Laboratory of Pordenone of Istituto Zooprofilattico Sperimentale delle Venezie for post mortem examination. The pig came from a farrow-to-weaning farm with a breeding herd of 550 sows.

The piglets received a vaccination against *Mycoplasma hyopneumoniae* (*M. hyopneumoniae)* and PCV2 at 28 days of life. In this farm, previous screening performed in weaned animals with respiratory signs revealed the presence of *M. hyorhinis* in broncho-alveolar lavage fluid (BALF). Moreover, clinical signs of enzootic pneumonia by day 150 of life are frequently recorded and, in 2018, lung scoring in slaughtered animals reported the frequent occurrence of cranioventral pulmonary consolidation. Finally, the breeding herd is PRRSV positive.

In the episode detailed herein, the farmer reported retarded growth since late lactating phase in 7% of piglets belonging to the last three farrowing batches. In addition, respiratory signs appeared in these piglets after weaning. Even if a treatment with macrolides was performed in affected suckling piglets, those who survived were still characterized by a reduced growth rate and the mortality rate after weaning (45–50 days of age) was around 3.5%.

The subject examined was characterized by reduced growth and did not receive any antimicrobial therapy within the last 20 days.

**Fig. 1:**
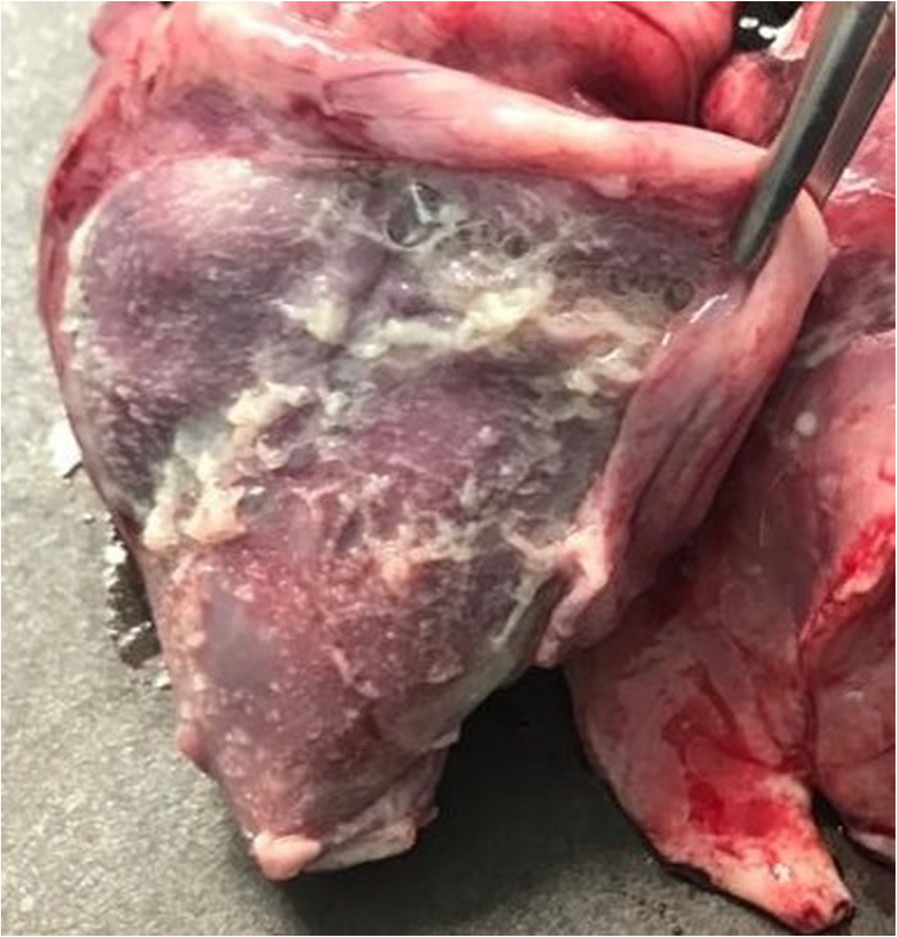
Fibrinous pericarditis

Macroscopically, the main finding was a fibrinous polyserositis with serious involvement of the pericardium which was opaque, thickened and contained abundant non-organized fibrin (Fig. 1). Other lesions included multifocal pleural adhesions, mild catarrhal bronchopneumonia, moderately enlarged mesenteric lymph nodes and mild increase in volume of the articular fluid in carpal and tarsal joints.

Swabs from pericardial and carpal fluid, lung and bronchus epithelium, were submitted for bacteriological investigation. Bacteriological procedures included blood agar and Eosin Methylen Blue agar incubated aerobically. In addition, blood agar with *Staphylococcus aureus* nurse culture incubated under microaerophilic condition was performed to allow growing of *Glaessarella parasuis (G. parasuis*) and *Actinobacillus pleuropneumoniae* (*A. pleuropneumoniae)*. All agar plates were incubated at 37 °C for 24–48 h. Finally, PCR for *G. parasuis* and *M. hyorhinis* was performed on pericardial and carpal fluid.

Bacteriological investigations allowed the isolation of *G. parasuis* from the lung, while bronchus, pericardium and carpal fluid samples were negative.

PCR was negative for *G. parasuis* and positive for *M. hyorhinis* (Ct = 19.5) in the pericardial fluid, while carpal fluid tested negative for both these pathogens.

Given the PCR positivity for *M. hyorhinis*, the pericardial fluid was then submitted also for *Mycoplasma* spp. culture. Both FRIIS broth and agar [10] were used as media and isolation of a pure culture of *M. hyorhinis* was obtained.

Antimicrobic susceptibility profile of the isolate was verified by MIC determination.

In the lack of official breakpoints the data were evaluated in comparison with other publications [6, 8, 15].

The MIC determination revealed that the strain was susceptible to florfenicol (0,5 μg/mL), oxytetracyclin (2 μg/mL), tiamulin (0,0625 μg/mL), spectinomycin (4 μg/mL) and resistant to enrofloxacin (4 μg/mL), erythromycin (> 32 μg/mL), lincomycin (> 32 μg/mL), spiramycin (16 μg/mL), tilmicosin (> 64 μg/mL), tylosin (32 μg/mL).

To rule out viral infections that could have predisposed the subject examined to bacterial diseases, PCR for PCV2, Influenza A Virus (IAV) and PRRSV was performed on the lung. Only PRRSV resulted positive.

Finally, to exclude concurrent intestinal infection, swabs from mesenteric lymph node and jejunum were submitted for bacteriology as detailed above. Two *Escherichia coli* (*E. coli*) strains were isolated, one haemolytic and one non-haemolytic. The haemolytic strain was tested for virulence genes and resulted positive for the fimbrial antigen F18 and the somatic antigen O139, while it tested negative for the following toxins coding genes: STX2e, LT, STI, STII.

In order to monitor and control this infection, an implementation of the screening tests on diseased and dead piglets (bacterial isolation, PCR and antimicrobic susceptibility profile by MIC) has been suggested to the farmer with the aims of verifying the prevalence, the potential multidrug resistances of this pathogenic strain and evaluating the best intervention approach.

## Discussion and conclusion

The most common agents of polyserositis in swine are *G. parasuis, Streptococcus suis* (*S. suis*) and *M. hyorhinis* [14].

Fibrinous pleuritis could also be induced by *A. pleuropneumoniae*, but, unlike this case, it is usually associated with focal, well demarcated, necrotic-hemorrhagic, solid areas in the lungs.

The most severe lesion found on this subject was the fibrinous pericarditis. As detailed above, our investigations included bacteriological and PCR test for the most common agents of polyserositis in swine, and *M. hyorhinis* was the only pathogen detected in the pericardial fluid. In fact, being isolated only form lung, *G. parasuis* was not considered significant for pericardial lesion.

The screening for the other common pathogens of swine (i.e. pathogenic *E. coli*, SIV, PRRSV and PCV2) evidenced the presence of PRRSV form the lung. It has to be considered that PRRSV can target immune cells and impair host defences, therefore it could have increased the susceptibility to opportunistic pathogens, such as *M. hyorhinis*.

The *E. coli* strain isolated from intestine and lacking virulence factor, is not to be considered pathogenetic.

Considering these results, the case described herein represents a fibrinous pericarditis due to *M. hyorhinis*.

*M. hyorhinis* is highly prevalent in domestic pig population as it is able to colonize the nasal cavity at an early age [2]. In addition, this bacterium is considered as one of the most common agent of polyserositis in Italian pig farms [14]. The diagnostic procedure includes the isolation of the microrganism, which requires both a specific culture medium and a prolonged incubation time. Therefore, It could be difficult to achieve and time consuming. Moreover, less fastidious and more common pathogens, like *G. parasuis* and *S. suis,* can cause similar gross lesions, and the co-infection is possible as well. Therefore, a complete and correct diagnostic approach is fundamental to reach the etiological diagnosis and to select the proper therapeutic protocol.

In this regard, autologous vaccines against *M. hyorhinis* have been reported by veterinary practitioners to be successful in reducing both lesions and clinical signs, thus, after strain isolation is achieved, the autologous vaccination could also represent a valid disease control measure [11, 12].

Data regarding *M. hyorhinis* antimicrobic susceptibility pattern are scarce, however, in agreement with a previous report [1, 16], the MIC profile of our strain shows high MIC values in a significant number of molecules, including the macrolides erythromycin, tylosin and tilmicosin. In respect to this, high MIC values for lincomycin have already been reported [13]. Additionally, the pleuromutilin tiamulin has been suggested as one of the most active antimicrobial in vitro against *M. hyorhinis* [5].

In conclusion, these findings underline the importance of achieving isolation and monitoring antimicrobial susceptibility profiles also for fastidious pathogens, like *M. hyorhinis,* in order to improve the control of this disease and to manage possible relapses of infection.

## Data Availability

Data sharing not applicable to this article as no datasets were generated or analysed during the current study.

## Abbreviations

MIC: Minimum Inhibitory Concentration
PCR: Polymerase Chain Reaction
